# A Real-World Prognosis in Idiopathic Pulmonary Fibrosis: A Special Reference to the Role of Antifibrotic Agents for the Elderly

**DOI:** 10.3390/jcm12103564

**Published:** 2023-05-19

**Authors:** Kojiro Honda, Takeshi Saraya, Haruyuki Ishii

**Affiliations:** Department of Respiratory Medicine, Kyorin University School of Medicine, Mitaka City 181-8611, Japan; h-kojiro78@ks.kyorin-u.ac.jp (K.H.); h141@ks.kyorin-u.ac.jp (H.I.)

**Keywords:** IPF, antifibrotic agents, survival probability, acute exacerbation, elderly patients, long-term treatment

## Abstract

Background: Idiopathic pulmonary fibrosis (IPF) is the most common and severe form of idiopathic interstitial pneumonia, and its prevalence increases with age. In the era of pre-antifibrotic agents, the median survival time of Japanese patients with IPF is 35 months, with a 5-year survival rate in western countries ranging from 20% to 40%. The prevalence of IPF is highest in elderly patients aged ≥75 years; however, the efficacy and safety of long-term use of pirfenidone and/or nintedanib are not fully understood. Objective: This study aimed to determine the efficacy and safety of the sole use of antifibrotic agents (pirfenidone or nintendanib) for IPF in the elderly. Method: We retrospectively reviewed patients with IPF who were diagnosed and treated with either pirfenidone or nintedanib in our hospital between 2008 and 2019. We excluded patients with the subsequent use of both antifibrotic agents. We examined the survival probability and frequency of acute exacerbation, with focus on long-term use (≥1 year), elderly patients (≥75 years of age), and disease severity. Results: We identified 91 patients with IPF (male to female ratio: 63 to 28, age 42 to 90 years). The numbers of patients with disease severity classified by JRS (I/II/III/IV) and GAP stage (I/II/III) were (38/6/17/20) and (39/36/6), respectively. The survival probabilities were comparable between the elderly (*n* = 46) and non-elderly groups (*n* = 45, *p* = 0.877). After the initiation of antifibrotic agents, the cumulative incidence ratio of acute exacerbation of IPF was significantly lower in the early stage (GAP stage I, *n* = 20) than in the progressive stage of disease (GAP stages II and III, *n* = 20, *p* = 0.028). A similar trend was noted in the JRS disease severity classification (I, II vs. III, IV) (*n* = 27 vs. *n* = 13, *p* = 0.072). In the long-term treatment (≥1 year) group (*n* = 40), the survival probabilities at 2 and 5 years after treatment initiation were 89.0% and 52.4%, respectively, which did not reach the median survival rate. Conclusions: Even in elderly patients (≥75 years of age), antifibrotic agents demonstrated positive effects on survival probability and the frequency of acute exacerbation. These positive effects would be improved for earlier JRS/GAP stages or long-term use.

## 1. Introduction

Idiopathic pulmonary fibrosis (IPF) is a specific form of chronic progressive fibrosing interstitial pneumonia of unknown cause that occurs in adults. Recently, Kondoh et al. reported that the nationwide number of IPF patients in Japan was estimated to be 34,040 (mean age: 73 years, percentage of men: 73%), and the prevalence was 27 per 100,000 people [1], which was higher than that of one prefecture (10.0 per 100,000 population) using clinical data (2003–2007) [2]. The prognosis of IPF is poor, with a median survival of 2–3 years if left untreated [3]. Moreover, acute exacerbation therapies [4] have a median survival rate following an AE of approximately 3–4 months [5]. Homma et al. reported that the annual incidence of acute exacerbation (AE) was approximately 10% between 2010 and 2018, based on a Japanese claims database (April 2008–March 2019) from acute care hospitals [6].

The use of antifibrotic agents (pirfenidone and nitedanib) can decrease the frequency of AE of IPF [7], even with short-term use in the perioperative phase [8]; however, little is known about the significance of antifibrotic agents in elderly patients (≥75 years of age) and their effect on survival in long-term use. The aim of this study is to clarify the efficacy and safety of antifibrotic agents (pirfenidone or nintendanib) for IPF in the elderly.

## 2. Materials and Methods

We retrospectively reviewed the medical records of patients with IPF who were treated with antifibrotic agents (pirfenidone or nintedanib) and visited our hospital between 2008 and 2019. Among the antifibrotic drugs, pirfenidone was administered at a daily dose of 600–1800 mg, while nintedanib was administered at a daily dose of 200 mg or 300 mg. IPF was diagnosed according to international guidelines [9]. High-resolution chest computed tomography was performed for diagnosis in all patients. This study excluded patients administered both antifibrotic medications. Clinical information obtained at the time of diagnosis included disease severity according to the Japanese Respiratory Society (JRS) classification or Gender, Age, and Physiology (GAP) stage, serum markers (Krebs von den Lungen-6 (KL-6) and surfactant protein D (SP-D)), respiratory function tests, age, sex, and body mass index. The definition of an elderly patient is over 75 years old, and we compared the findings between elderly (≥75 years of age) and non-elderly (≤74 years of age) patients. Clinical courses such as survival probability and acute exacerbation from the first visit to our hospital (at the time of diagnosis) and the timing of initial treatment with antifibrotic drugs to those events were also recorded.

### Statistical Analysis

Numerical data were evaluated for normal distribution and equal variance using Kolmogorov–Smirnov and Levene median tests, respectively. Categorical data are presented as percentages of the total or numerically, as appropriate. Statistical comparisons of non-parametric data were performed using Mann–Whitney U tests. Categorical data were compared using Pearson’s chi-square tests. All tests were two-sided, with statistical significance set at *p* < 0.05. Based on the extracted parameters, we performed Cox regression analysis, Kaplan–Meier analysis, and log-rank analysis. The data were analyzed using IBM SPSS Statistics for Windows, version 25.0. This study was approved by the ethics committee of Kyorin University Hospital (approval number: 968, H29-044).

## 3. Results

We enrolled 91 patients with IPF, comprising 46 elderly and 45 non-elderly patients. The male-to-female ratio was approximately 2:1. The patient ages ranged from 42 to 90 years, with median ages in the elderly and non-elderly groups of 79 and 68 years, respectively. Patient characteristics, such as age, sex, treatment with antifibrotic agents, and disease severity, based on JRS classification or GAP state, were comparable between the elderly and non-elderly groups (Table 1). According to the disease severity score, the patients with IPF were categorized according to the JRS classification (I/II/III/IV; 38/6/17/20) and GAP models (I/II/III; 39/36/6). Mild disease was predominantly observed in both the elderly and the non-elderly groups.

All data are presented as median (interquartile range). JRS classification and GAP stage include only the available data of 81 patients.

Similarly, the serum levels of KL-6, SP-D, and LDH and the results of respiratory function tests (%FVC, %DLco, and %DLco/VA) were comparable between the elderly and the non-elderly groups (Table 1).

### 3.1. Analysis of Survival Probabilities

#### 3.1.1. Survival Probabilities of All Enrolled Patients

**Figure 1:**
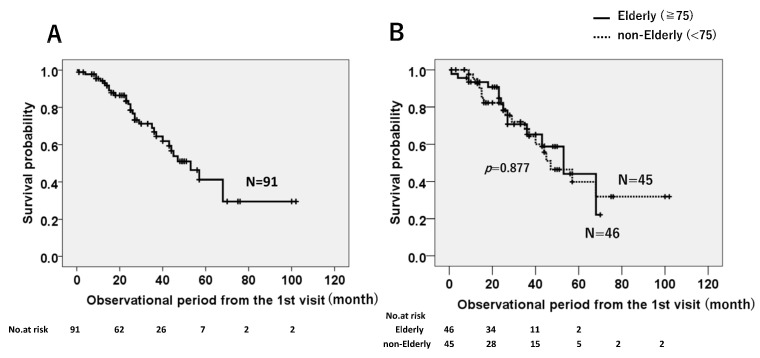
Survival probabilities based on the Kaplan–Meier method for all enrolled patients: (**A**) The median survival time (MST) from the first visit to our hospital was 53 months. (**B**) The MST did not differ between the elderly and non-elderly groups.

The Kaplan–Meier analysis showed a median survival time (MST) of all patients (*n* = 91) from the first visit to our hospital of 53 months (Figure 1A). The MSTs were comparable between the elderly (*n* = 46) and non-elderly (*n* = 45) groups (53 months vs. 47 months, *p* = 0.877) (Figure 1B).

#### 3.1.2. Survival Probabilities according to Treatment Regimen

**Figure 2:**
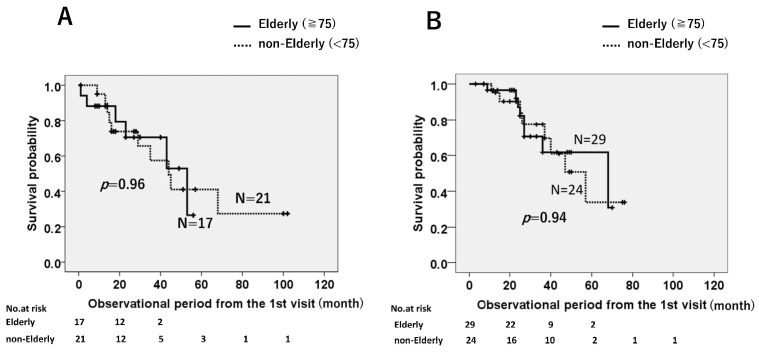
Survival probabilities according to the Kaplan–Meier method for patients treated with pirfenidone (**A**) and nintedanib (**B**), showing that the mean survival times (MSTs) did not differ between elderly and non-elderly groups in each treatment group.

Kaplan–Meier analysis of patients administered pirfenidone showed no difference in MST between the elderly (*n* = 17) (40.3 months) and non-elderly (*n* = 21) (53.2 months, *p* = 0.96) groups (Figure 2A). A similar trend was observed for nintedanib-treated patients: elderly (*n* = 30) (51.0 months) vs. non-elderly (*n* = 24) (52.6 months, *p* = 0.94) (Figure 2B).

#### 3.1.3. Survival Probabilities of All Enrolled Patients (*n* = 91) according to the Status of Successful 1-Year Treatment

**Figure 3:**
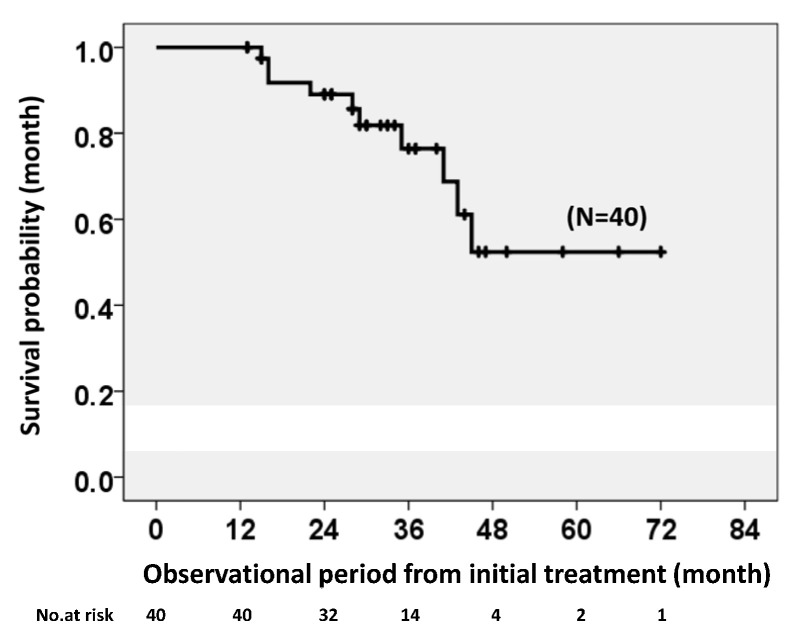
Survival probabilities after treatment among patients with IPF and antifibrotic agent use for ≥1 year. The 2- and 5-year survival probabilities (89.0% and 52.4%, respectively) did not reach the median survival rate.

Kaplan–Meier analysis showed the survival probabilities of patients with long-term treatment of IPF (*n* = 40) at 2 and 5 years after initial treatment of 89.0% and 52.4%, respectively, which did not reach the median survival rate (Figure 3).

### 3.2. Cumulative Incidence Ratios of Acute Exacerbation of IPF

#### 3.2.1. Cumulative Incidence Ratio of Acute Exacerbation of IPF with Long-Term Antifibrotic Agent Use (>1 year) Based on JRS or GAP Stages

**Figure 4:**
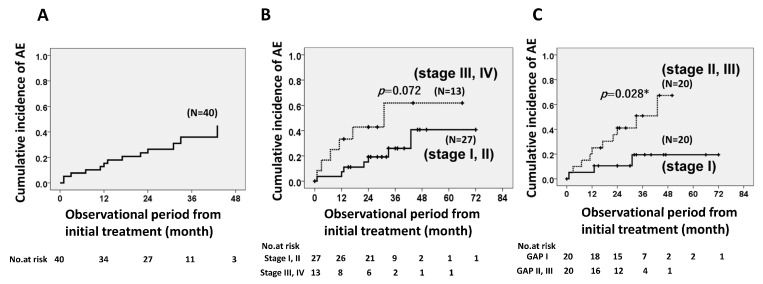
Cumulative incidence ratios of acute exacerbation of IPF. The ratios within 1, 2, and 3 years among patients with long-term antifibrotic agent use (≥1 year, *n* = 40) were 15.4%, 26.3%, and 36.0%, respectively (**A**). Patients with mild disease (JRS classifications I and II) showed a lower occurrence of AE compared to those with moderate to severe stages (classes III and IV) (**B**). Patients with GAP stage I showed a significantly lower incidence of AE compared to those with GAP stages II and III during the observational period (**C**). * means *p* value is less than 0.05.

Among patients with >1 year of treatment (*n* = 40), the cumulative incidence ratios of AE of IPF within 1, 2, and 3 years were 15.4%, 26.3%, and 36.0%, respectively (Figure 4A). According to disease severity, mild disease based on JRS classifications I and II (*n* = 27) showed a lower occurrence of AEs compared to classes III and IV (*n* = 13) during the observational period; however, the difference was not statistically significant (*p* = 0.072) (Figure 4B). In contrast, the incidence of AE was significantly lower for GAP stage I (*n* = 20) compared to GAP stages II and III (*n* = 20, *p* = 0.028) during the observational period (Figure 4C).

#### 3.2.2. Cumulative Incidence Ratio of AE of IPF with Long-Term Antifibrotic Agent Use (>1 year) according to the Type of Antifibrotic Agent

Patient characteristics were comparable in terms of sex, age, body mass index, disease severity according to JRS classification or GAP stage, and serum data (except for the %DLco/VA) when comparing between the pirfenidone and nintedanib groups (Table 2).

**Figure 5:**
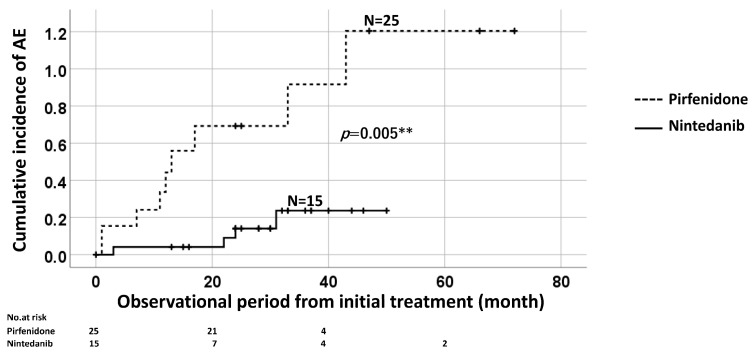
Log-rank analysis showed a significantly higher cumulative incidence of AE in the pirfenidone group than that in the nintedanib group. ** means *p* value is less than 0.01.

In the log-rank analysis, the cumulative incidence of AE was significantly higher in the pirfenidone group (*n* = 25) than that in the nintedanib group (*n* = 15) (*p* = 0.005) (Figure 5). The cumulative incidences of AE within 1, 2, and 3 years in the pirfenidone and nintedanib groups were (35.7%, 50%, and 60%) and (4%, 13.1%, and 21%), respectively.

On simple cox regression analysis, use of pirfenidone was associated with a significantly higher risk for AE than that of nintedanib (hazard ratio (HR) 4.757, 95% confidence interval (CI) 1.449–15.613, *p* = 0.01). The risk of AE was lower in females than in males (HR 0.096, HR 0.011–0.829, *p* = 0.033). On the other hand, “age” and/or “elderly itself” cannot be significant parameters for predicting AE.

Multiple cox regression analysis showed that the use of pirfenidone was associated with a significantly higher risk of AE compared to nintedanib (HR 5.840, 95% CI 1.311–26.007, *p* = 0.021), while the risk of AE was lower in female than in male patients (HR 0.091, 95%CI 0.009–0.963, *p* = 0.046). Age was not a predictive factor for AE (HR 0.974, 95% CI 0.9.9–1.043, *p* = 0.450).

### 3.3. Effects of Antifibrotic Agents in Elderly Patients with IPF

Among all elderly IPF patients, the durations from the first visit to initial treatment did not differ significantly between the >1-year and <1-year treatment groups (median 2.0 months, IQR: 0–15.0 months vs. median 6.5 month, IQR: 1.8-–13.3 months, *p* = 0.193) groups (Table 3). Similarly, the timings for the initiation of treatment with pirfenidone (median 4 months, IQR: 0–11 months) and nintedanib (median 1 month, IQR: 1–16.5 months, *p* = 0.174) did not differ significantly.

**Figure 6:**
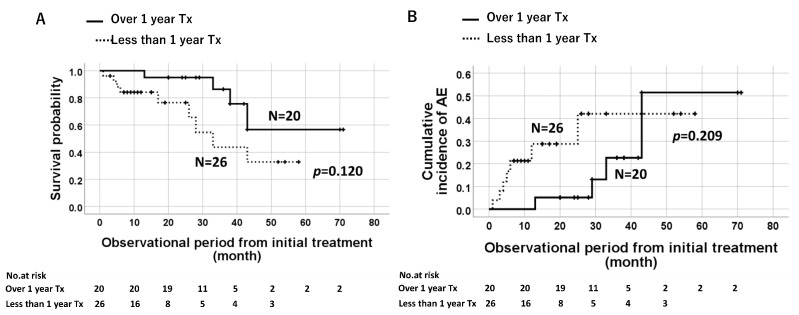
Log-rank analysis showing comparable survival probabilities between longer (≥1 year) and shorter (<1 year) treatment durations (**A**). The cumulative incidence of AE is also comparable between two groups (**B**).

**Figure 7:**
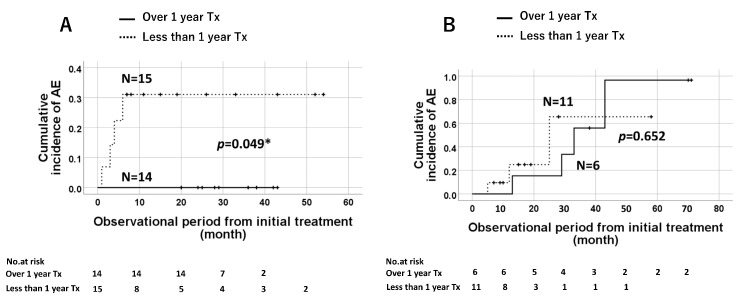
Treatment-based analysis in elderly patients with IPF. The cumulative incidence of acute exacerbation (AE) in patients administered nintedanib is significantly lower in those with prolonged treatment duration (≥1 year) compared to those with a shorter treatment duration (**A**). This trend was not observed for pirfenidone treatment (**B**). * means *p* value is less than 0.05.

The Kaplan–Meier analysis of elderly patients with IPF (*n* = 46) showed that prolonged treatment (>1 year) did not affect the survival probability (Figure 6A), the cumulative incidence of AE (Figure 6B), or the total AE. However, according to the type of antifibrotic agents, the incidence of AE was significantly lower in the groups of patients administered nintedanib for >1 year compared to that in patients administered the drug for <1 year (Figure 7A, *p* = 0.049). This trend was not observed for pirfenidone treatment (Figure 7B).

Cox regression analysis showed that pirfenidone use (HR 2.890, 95% CI 0.845–9.886, *p* = 0.091) or sex (female) (HR 0.200, 95% CI 0.025–1.565, *p* = 0.125) were not predictive factors for AE.

The frequencies of adverse effects were comparable between groups administered treatment for >1 and <1 year (Table 3). The adverse events mainly consisted of appetite loss, diarrhea, liver dysfunction, and dysgeusia. Among all adverse effects, grade III or higher severity was observed in only one patient with diarrhea, who discontinued treatment (Table 3). Similarly, the proportions of patients discontinuing and/or reducing the dose of antifibrotic agents did not differ significantly according to treatment duration.

## 4. Discussion

Although the effects of antifibrotic agents on the prognosis of patients with IPF remain unclear, the results of this study provided real-world evidence that antifibrotic agents prolonged the survival times of patients with IPF compared to previous reports (2, 3), and also showed the beneficial effects even in elderly patients (≥75 years of age). Moreover, the long-term use (>1 year) of antifibrotic agents also showed positive effects on survival probability compared to short-term use (<1 year). This effect was probably due to a decrease in the cumulative incidence of AE, which will enhance the early initiation of antifibrotic agents in mild-stage IPF (JRS classification I and II or GAP stage I).

Natsuizaka et al. [2] reported an MST of patients with IPF patients in Japan of 35 months, while foreign countries reported MSTs ranging from 3 to 5 years [10]. Our real-world data demonstrated that the use of antifibrotic agents prolonged the MST (53 months, calculated from the first visit to our hospital), and did not reach the MST (calculated from the day of initial treatment), suggesting the importance of continuous treatment.

Kang et al. [11] reported that antifibrotic treatment reduced the risks of all-cause mortality, hospitalization (all-cause and respiratory-related), AE, and mortality after AE, but did not refer to the duration of treatment. Dempsey et al. [12] reported that the risk of mortality or hospitalization decreased only during the first 2 years of antifibrotic treatment. Although the most important known prognostic determinants for mortality are declined lung function, acute exacerbations, and pulmonary hypertension, we found that long-term antifibrotic agent use (>1 year) decreased the incidence of AE, which resulted in increased survival probabilities.

We also found that the initiation of antifibrotic agents in early stage IPF accelerated the beneficial effects of AE to prevent a decline in lung function or risk of mortality. Sugino et al. [13] also reported the beneficial effects of antifibrotic agents on %FVC for patients with JRS stage I with oxygen desaturation on the 6 minutes’ walk test or increased GAP staging (II/III). Similarly, Poletti et al. [14] described that the early diagnosis and prompt initiation of antifibrotic therapy for IPF patients preserved lung function 12 months after baseline. Their study enrolled patients with relatively mild-stage IPF, with a mean predicted FVC% of 80.01%, suggesting the importance of the early induction of antifibrotic agents in mild stages. Another study [15] demonstrated the beneficial effect of antifibrotic agents on survival probability in patients with GAP stage > 1, with higher probabilities observed for nintedanib compared to pirfenidone.

In this study, among the 40 patients with long-term treatment, nintedanib showed a more beneficial effect on AE compared to pirfenidone, which might be due to the low DLco/VA at enrollment in the pirfenidone group. However, the beneficial effects on AE were observed even in elderly patients with IPF with prolonged nintedanib use (>1 year). Isshiki et al. [7] reported a significantly lower cumulative incidence of AE for pirfenidone compared to nintedanib. Thus, our data should be cautiously interpreted and confirmed in further prospective studies with larger numbers of patients. Previous studies reported that both nintedanib and pirfenidone can preserve lung function or reduce the risk of mortality compared to non-treated patients [16,17,18,19]. Furthermore, Cameli et al. [20] reported similar effectiveness between pirfenidone and nintedanib in terms of mortality and functional disease progression. However, which drug should be preferred for early initiation in the mild stage is an unresolved critical issue.

This study had several limitations. First, this was a retrospective observational study with a relatively small number of IPF patients. Second, this study was performed at a single center. These factors may limit the generalizability of our results; however, we eliminated patients treated with both nintedanib and pirfenidone and patient management was less variable than that in multicenter cohorts. Third, this study did not describe in detail the adverse events associated with antifibrotic agents. Uchida et al. [21] reported that grade 2 nausea, diarrhea, and emaciation were the most common adverse events of antifibrotic agent use in patients aged ≥ 75 years, while grade 4 was not. The same trend was observed in this study.

This study provided real-world data demonstrating the positive effects of antifibrotic agents use on the survival probability and frequency of AE even in elderly patients. These effects were greater in early stage IPF based on JRS/GAP or with long-term use, which requires confirmation in future studies.

## Figures and Tables

**Table 1:** Patient characteristics.

	All Patients (*n* = 91)	Elderly Group (*n* = 46)	Non-Elderly Group (*n* = 45)	*p*-Value
Gender (male/female)	63/28	32/14	31/14	1.0
Age	75 (68–79)	79 (76–81)	68 (63–72)	<0.001 ***
Comorbidity				
Malignancy	17	10	7	0.592
Cardiovascular disease	7	2	5	0.267
Diabetes mellitus	15	9	6	0.574
COPD	6	2	4	0.434
JRS classification (I/II/III/IV)	(38/6/17/20)	(17/5/6/10)	(21/1/11/10)	1
GAP stage (I/II/III)	(39/36/6)	(16/22/2)	(23/14/4)	0.141
Duration from 1st visit to initial therapy (month)	6 (1–17)	4 (1–13)	8 (3–25)	0.123
Pirfenidone/Nintedanib	37/54	16/30	21/24	0.29
KL-6 (IU/mL)	1004 (644–1745)	816 (500–1957)	868 (465–1342)	0.948
SP-D (ng/mL)	249 (165–358)	207 (142–388)	209 (143–353)	0.378
BMI	23.4 (20.9–25.9)	22.8 (21.1–24.9)	24.9 (21.7–28.6)	0.07
Alb (g/dL)	4.1 (3.9–4.4)	4.1 (3.8–4.4)	4.2 (3.9–4.4)	0.238
LDH (U/mL)	233 (201–262)	222 (201–254)	215 (197–265)	0.984
%FVC	75.2 (61.3–88.3)	76.8 (61.0–85.5)	81.8 (58.4–94.5)	0.824
%DLco	54.1 (40.8–71.8)	55.4 (41.5–70.1)	57.7 (40.8–80.9)	0.898
%DLco/VA	69.7 (48.5–83.8)	68.9 (53.1–83.5)	76.8 (51.2–90.6)	0.501

*** means *p* value is less than 0.001.

**Table 2:** Patient characteristics of successful long-term antifibrotic treatments.

	All Patients (*n* = 40)	Pirfenidone (*n* = 15)	Nintedanib (*n* = 25)	*p*-Value
Gender (male/female)	28/12	10/5	18/7	0.736
Age	75 (67–79)	68 (56–75)	76 (67–81)	0.737
BMI	23.9 (22.3–26.9)	24.9 (23.8–43.6)	23.3 (21.2–26.4)	0.371
JRS classification (I–II/III–IV)	(27/13)	(8/7)	(19/6)	0.175
GAP stage (I/II–III)	(20/20)	(6/9)	(14/11)	0.514
Duration from 1st visit to initial treatment (month)	6.0 (1.0–16.0)	6.0 (0–19.0)	6.0 (2.0–15.0)	0.510
KL-6 (IU/mL)	956 (576–1445)	748 (395–1479)	868 (540–1186)	0.410
SP-D (ng/mL)	209 (142–311)	146 (138–323)	191 (137–266)	0.808
Alb(g/dL)	4.1 (3.9–4.3)	4.1 (3.8–4.3)	4.0 (3.9–4.4)	0.676
LDH (U/mL)	217 (198–246)	208 (187–234)	212 (200–235)	0.806
%FVC	73.4 (61.7–87.5)	81.8 (53.6–93.8)	77.4 (60.3–103.8)	0.530
%DLco	55.5 (46.5–70.1)	62.8 (51.2–77.7)	58.9 (48.0–70.2)	0.365
%DLco/VA	76.7 (52.5–86.3)	70.9 (46.1–94.2)	80.9 (63.8–89.9)	0.038 *

All data are presented as median (interquartile range). * means *p* value is less than 0.05.

**Table 3:** Characteristics of elderly patients with or without long-term treatment with antifibrotic agents.

	Over 1 Year Tx	Less than 1 Year Tx	*p* Value
Number of patients	20	26	
Male	12	19	0.333
Age (years)	79.5 (76.0–81.0)	78.0 (77.0–79.3)	0.180
JRS classification (Ⅰ/Ⅱ/Ⅲ/Ⅳ)	9/3/1/5	10/3/5/5	0.561 ^a^
GAP stage (Ⅰ/Ⅱ/Ⅲ)	7/13/0	8/18/0	1.0 ^b^
Comorbidity			
Malignancy	3	7	0.476
lung cancer	1	1	
prostate cancer	1	2	
lymphoma	1	0	
rectal cancer	0	1	
gastric cancer	0	1	
renal cell cancer	0	1	
unknown	0	1	
Cardiovascular disease	0	2	0.498
Diabetes mellitus	5	4	0.472
COPD	0	2	0.498
Pirfenidone/Nintedanib	6/14	11/15	0.534
Duration of antifibrotic Tx (months)	24.5 (18.0–34.3)	5.0 (2.3–7.8)	0.001
AE	4	7	0.728
Number of AEs after Tx			
Duration of initial treatment to AE (months)	34.5 (24.3–41.0)	13.5 (7.0–26.5)	<0.001
Adverse effects	5 (1)	8 (0)	0.749
appetite loss	2	4	0.684
diarrhea	2 (1)	3	1.0
liver dysfunction	1	0	0.435
dysgeusia	0	2	0.498
Discontinuation of Tx	3	5	1.0
Dose reduction of Tx	2	3	1.0

All data are presented as median with interquartile range. Both “over 1 year Tx” and “less than 1 year Tx” groups have unknown cases (*n* = 2 and *n* = 3) for JRS classification. “^a^” means JRS classification I/II vs. III/IV, “^b^” means GAP stage I vs. II/III. AE: acute exacerbation, Tx: therapy, parentheses means number of grade 3. JRS: Japanese respiratory society. GAP: Gender, Age, and Physiology. COPD: chronic obstructive pulmonary disease.

## Data Availability

The data used and/or analyzed in this study are available from the corresponding author on reasonable request. All data generated or analyzed during this study are included in this manuscript, and the database is available from the corresponding author (sara@yd5.so-net.ne.jp) upon reasonable request.
